# Automated coronary artery calcium scoring in patients with breast cancer to assess the risk of heart disease following adjuvant radiation therapy

**DOI:** 10.1016/j.breast.2022.07.003

**Published:** 2022-07-11

**Authors:** Kangpyo Kim, Seung Yeun Chung, Caleb Oh, Iksung Cho, Kyung Hwan Kim, Hwa Kyung Byun, Hong In Yoon, Jaewon Oh, Jee Suk Chang

**Affiliations:** a Department of Radiation Oncology, Yonsei Cancer Center, Yonsei University College of Medicine, Republic of Korea; b Department of Radiation Oncology, Ajou University Hospital, Ajou University School of Medicine, Republic of Korea; c Cardiology Division, Severance Cardiovascular Hospital and Cardiovascular Research Institute, Yonsei University College of Medicine, Republic of Korea; d Department of Radiation Oncology, Gangnam Severance Hospital, Yonsei University College of Medicine, Republic of Korea

**Keywords:** Acute coronary events, Breast cancer, Coronary artery calcium scores, Radiotherapy, Risk factors

## Abstract

**Aim:**

Validation of coronary artery calcium (CAC) scores as prognostic factors of acute coronary events (ACE) development in breast cancer patients are demanded. We investigated prognostic impact of CAC on ACE development with cardiac exposure to radiation.

**Methods:**

We evaluated breast cancer patients with (n = 511) or without (n = 600) adjuvant radiotherapy (RT) between 2005 and 2013. CAC Agatston scores were analyzed using a deep-learning-based algorithm. Individual mean heart dose (MHD) was calculated, and no RT was categorized as 0 Gy. The primary endpoint was the development of ACE following breast surgery.

**Results:**

In the RT and no-RT cohorts, 11.2% and 3.7% exhibited CAC >0, respectively. Over a 9.3-year follow-up period, the 10-year ACE rate was 0.7%. In the multivariate analysis, the CAC score was a significant risk factor for ACE (CAC >0 vs CAC = 0, 10-year 6.2% vs 0.2%, P < 0.001). In the subgroup with CAC >0, the 10-year ACE rates were 0%, 3.7%, and 13.7% for patients receiving mean heart doses of 0 Gy, 0–3 Gy, and >3 Gy, respectively (P = 0.133). Although CAC score was not predictive for non-ACE heart disease risk (P > 0.05), the 10-year non-ACE heart disease rates were 1.7%, 5.7%, and 7.1% for patients with CAC = 0 receiving MHD of 0 Gy, 0–3 Gy, and >3 Gy, respectively (P < 0.001).

**Conclusions:**

The CAC score was a significant predictor of ACE in patients with breast cancer. Although further studies are required, CAC score screening on simulation CT in patients undergoing breast RT can help identify those with high risk for ACE on a per-patient basis.

## Highlights

• CAC score was successfully validated as a strong predictive factor for ACEs.
• MHD was identified as a significant factor in development of ACE and NAHD.
• Best efforts should be made to keep the dose to cardiac structures as low as possible.

## 1 Introduction

Radiotherapy (RT) is widely used in breast cancer treatment to prevent a locoregional recurrence and improve survival [1]. In the modern treatment era, the risk of radiation-induced heart disease (RIHD) is of increasing concern among breast cancer survivors [2,3]. Among RIHD, acute coronary events (ACEs) have drawn much attention, and well-known dosimetric risk factors for ACEs are the mean heart dose (MHD), the left ventricle volume receiving 5 Gy radiation (LV-5) and dose to the left anterior descending coronary artery [[4], [5], [6], [7]].

One notable risk factor for ACEs development in the general population is high coronary artery calcium (CAC) score, which is represented as an Agatston score (AS) [[8], [9], [10]], and it is known to be beneficial to use prophylactic agents in patients with a CAC score >0 [11,12]. Recently, the application of a deep-learning software successfully reduced the time and labor required for manual CAC scoring, and a wider application of the scoring system was possible for selecting high-risk patients with ACE development among the general population [13,14]. In breast cancer patients, the CAC score was shown to be ACEs' predictive risk factor [[15], [16], [17]]; however, it remains unclear whether the CAC score and radiation exposure interact synergistically to exacerbate the ACEs’ risk.

According to recent findings from Western studies on patients with breast cancer, the CAC score has shown to be predictive in the development of ACE in breast cancer patients [15,18]. However, the value of the CAC score needs to be confirmed and validated in other geographic areas as cardiac diseases have a multifactorial etiology, including race- and patient-specific factors. Therefore, this study tried to validate the CAC score's prognostic impact on development of ACEs. We also attempted to identify the interaction between CAC score and radiation exposure in ACE development by comparing the patients who received or did not receive RT.

## 2 Materials and methods

### 2.1 Patients

We used a dataset of 1294 patients from a previous study [4] in which 1111 patients with non-electrocardiogram (ECG) synchronized CT scans were analyzed. Patients without non-contrast CT or PET-CT to calculate CAC score were excluded from the analysis. The MHD of the patients who did not receive RT was defined as a 0 Gy dose. This study was approved by the Institutional Review Board of Severance hospital (IRB no. 4-2021-1342).

### 2.2 RT regimens

All RT plans for the patients were based on CT-based simulations. Using 4–6-MV X-ray linear accelerators, 50.4 Gy dose at 1.8 Gy per fraction was applied to the whole breast or chest wall, with or without the regional nodal area. Comprehensive regional node irradiation (RNI) involving the supraclavicular (SCL), axillary level I-III, and internal mammary lymph nodes (IMN) was performed in patients with positive lymph node metastasis or node-negative disease with multiple adverse features, including medial tumor localization. Regarding the radiation procedures, a tangential field technique was used for patients who received whole breast irradiation. A “reverse hockey stick” field with a custom-made step bolus was used to cover the whole regional lymph node area, including the SCL, axillary, and IMN, employing a moving junction technique at 25.2 Gy to improve dose homogeneity. A three-field monoisocentric and partial wide tangent technique was used when the IMN were excluded from the RT target area. Tumor bed boost RT after breast-conserving surgery was performed using an electron beam at 10 Gy delivered in five fractions. We delineated the heart according to the cardiac atlas [19], and MHD was extracted from a clinical report from the MIM software (MIM Software Inc, Cleveland, OH).

### 2.3 CAC scoring

CAC scores were determined automatically using a deep-learning-based calcium scoring algorithm (AVIEW-CAC, v1.1.38.6, Coreline Soft, Seoul, Korea). The AVIEW-CAC software used in our study was trained using 1) an asymptomatic population who underwent health check-up (screening group; n = 2653) [20]; 2) symptomatic patients who underwent invasive fractional flow reserve measurements (FFR group; n = 222) [21]; and 3) patients with mitral valve prolapse who underwent preoperative coronary CT angiography (valve group, n = 145) [22]. All CT scans used in the training were performed in the prospective ECG triggering or retrospective ECG gating mode. We used RT-planning CT scans (from patients who received RT) and PET-CT scans (from patients who did not receive RT) without ECG synchronization, which were examined around 6 months after the breast cancer diagnosis. Supplementary Fig. 1 demonstrates the AVIEW-CAC software’s automatic CAC scoring procedure. The calcium scoring technique's statistical reproducibility was shown to be high, with a linearly weighted kappa value of 0.91 [13]. The performance of the AVIEW-CAC software was successfully validated in external dataset of non-ECG-gated CT scans and was further updated for our analysis (Currently under submission). Here, the AS was used in the analyses, as that is the score that has been most commonly used in previous studies.

### 2.4 Follow-up evaluations

Follow-up evaluations comprised physical examinations, radiologic imaging studies, and laboratory tests conducted every 6 months for 5 years and then annually thereafter. The disease spectrum of ACEs included ST-elevation/non-ST-elevation myocardial infarction and unstable angina pectoris, which were confirmed through coronary angiography. The spectrum of non-ACE heart disease (NAHD) included any heart toxicity except ACE, which is described in the Common Terminology Criteria for Adverse Events ver 5.0. All ACEs and NAHDs were independently reviewed by certified cardiologists in a blinded fashion based on the current European Society of Cardiology guidelines.

### 2.5 Statistical analysis

This study's primary endpoint was ACE development following breast surgery. The cumulative probabilities of ACEs were calculated using Kaplan-Meier survival. These probabilities were compared using the log-rank test.

We first investigated whether the baseline CAC score was associated with post-treatment ACE risk in patients with or without breast RT. Second, we conducted a subgroup analysis according to breast RT status to test the hypothesis that the calcium plaques' presence in coronary arteries is an important factor driving the radiation-related ACEs development. Lastly, the dose-response relationship was investigated. We subsequently assessed and visualized the correlations between baseline CAC locations, corresponding irradiation doses, and the stenosis areas in each ACE.

Patient characteristics were compared using chi-squared tests. For multivariate analysis, Cox regression model was used including variables with P < 0.1 in univariate analysis. In order to reduce the problem of overfitting as much as possible for multivariate analysis, the available baseline risk factors which were previously defined by Darby et al., were included as a dichotomous variable (none vs one or more risk factors) [23]. All analyses were performed using SPSS version 23.0 (IBM Inc, Armonk, NY) and R version 3.5.1. (R Foundation for Statistical Computing, Vienna, Austria) software.

## 3 Results

### 3.1 Patient characteristics

Table 1 shows the patient characteristics for the entire cohort.

**Table 1:** Patient and disease characteristics (N = 1111).

Variables	Whole cohort (N=1111)		RT (N=511)		no RT (N=600)		p value
	No.	%	No.	%	No.	%	
**Age (Years)**	Median 50 (range, 24–87)		Median 51 (range, 27–83)		Median 50 (range, 24–87)		0.393

**Median follow up years (range)**	Median 9.3 years (range 4.9–15.6)		Median 8.8 (range, 4.9–15.6)		Median 10.8 (range, 5.1–14.8)		

**Disease laterality**							0.477
Left	615	55.4	277	54.2	338	56.3	
Right	496	44.6	234	45.8	262	43.7	
**BMI (Kg/m**^**2**^**)**	Median 22.86 (range, 14.6–35.9)		Median 23.4 (range, 14.6–34.7)		Median 22.8 (range, 16.0–35.9)		0.776

**Exercise hx**							0.001
Yes	176	15.8	100	19.6	76	12.7	
No	935	84.2	411	80.4	524	87.3	
**Heart disease hx**							0.548
Yes	23	2.1	12	2.3	11	1.8	
No	1088	97.9	499	97.7	589	98.2	
**Smoking hx**							0.622
Current-smoker	10	0.9	4	0.8	6	1.0	
Ex-smoker	17	1.5	6	1.2	11	1.8	
Non-smoker	1084	97.5	501	98	583	97.2	
**HTN**							0.832
Yes	249	22.4	116	22.7	133	22.2	
No	862	77.6	395	77.3	467	77.8	
**DM**							0.03
Yes	74	6.7	43	8.4	31	5.2	
No	1037	93.3	468	91.6	569	94.8	
**Type of surgery**							<.001
PM	408	36.7	407	79.6	1	0.2	
MRM	703	63.3	104	20.4	599	99.8	
**Anthracycline use**							0.008
Yes	613	55.2	304	59.5	309	51.5	
No or other CTx	498	44.8	207	40.5	291	48.5	
**Anti-HER2 treatment**							0.013
Yes	126	11.3	71	13.9	55	9.2	
No	985	88.7	440	86.1	545	90.8	
**Tumor bed boost**							
**Yes**	399	35.9	399	78.1			
**No**	712	64.1	112	21.9			
**RNI**							
Yes	227	20.4	227	44.4			
No	884	79.6	284	55.6			
**RT total dose (Median, Range)**			59.4 Gy (range, 42.6 Gy–66.4 Gy)		0 Gy		

**Mean heart dose (Median, Range)**			3.4 Gy (range, 0 Gy–14.16 Gy)		0 Gy		

*Abbreviations: RT; radiotherapy, BMI; body mass index, hx; history, HTN; hypertension, DM; diabetes mellitus, PM; partial mastectomy, MRM; modified radical mastectomy; CTx; chemotherapy, HER2; human epidermal growth factor receptor 2, RNI; regional lymph-node irradiation (axillary lymph node level 1 to 3, supraclavicular lymph node, and internal mammary lymph node).

Of the 1111 breast cancer patients, 511 received RT. The median follow-up times were 8.8 years (range, 4.9–15.6 years) and 10.8 years (range, 5.1–14.8 years) for the RT and no-RT groups, respectively. The RT group comprised significantly more patients with DM or those who received anthracycline-based chemotherapy than the no-RT group, and the number of patients with regular exercise was also higher in the RT group.

Patient characteristics between RT and no-RT group were also compared in those with CAC score >0, and there were no significant differences.

### 3.2 ACEs and other types of heart disease

There were seven patients with ACE and 44 with NAHD. All events were non-fatal, and the median f/u period after the development of ACE and NAHD were 82.5 months (range, 24–104 months), and 63 months (range, 16–123 months), respectively.

**Fig. 1:**
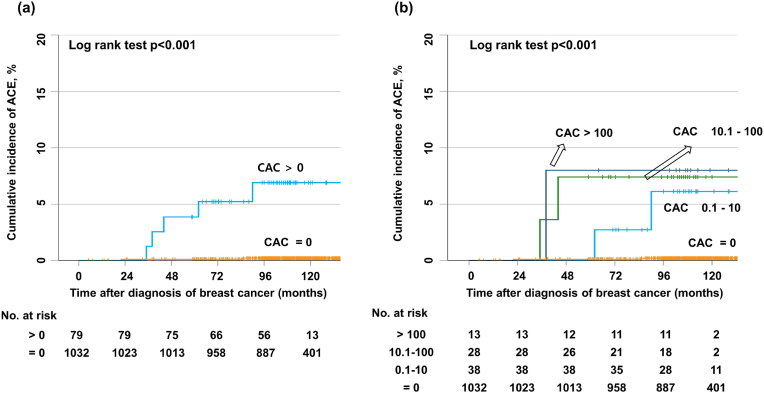
Cumulative incidence of acute coronary events (ACEs) in patients categorized into two groups with a coronary artery calcium (CAC) score = 0 or >0 (a). Cumulative incidence of ACEs in patients categorized into four groups with a CAC score = 0, 0.1–10, 10.1–100, and >100 (b).

The ACEs' 10-year cumulative incidence in the entire cohort was 0.7% over a 9.3-year median follow-up period (interquartile range, 8.4–10.3 years). Patients with a CAC score >0 experienced significantly more ACEs than patients with a CAC score = 0 (10-year incidence of 6.7% vs 0.2%, respectively; Fig. 1a). According to the CAC scores' further stratification (0, 0.1–10, 10.1–100, and >100), the ACEs’ incidence increased with increasing CAC scores (Table 2, Fig. 1b).

**Table 2:** Total CAC score (Agatston score) and cumulative incidence of ACE.

Variables	N (%)	ACE event	5 year incidence	10 year incidence	p-value
**CAC score (Total, N** = **1111)**					<0.001
0	1032 (92.9)	2	0.1%	0.2%	
0.1–10	38 (3.4)	2	2.7%	5.9%	
10.1–100	28 (2.5)	2	7.1%	7.1%	
>100	13 (1.2)	1	7.7%	7.7%	
**CAC score (RT, N** = **511)**					<0.001
0	454 (88.8)	2	0.2%	0.5%	
0.1–10	23 (4.5)	2	4.3%	9.1%	
10.1–100	25 (4.9)	2	8.0%	8.0%	
>100	9 (1.7)	1	11.1%	11.1%	
**CAC score(no RT, N** = **600)**					N/A
0	578 (96.3)	0	0.00%	0.00%	
0.1–10	15 (2.5)	0	0.00%	0.00%	
10.1–100	3 (0.5)	0	0.00%	0.00%	
>100	4 (0.7)	0	0.00%	0.00%	

*Abbreviations: CAC; coronary artery calcium, ACE; acute coronary event, RT; radiation therapy.

In the multivariate Cox regression analysis, the significance of a CAC score >0 was retained, and MHD over 3 Gy was also identified as a risk factor for ACEs (Table 3).

**Table 3:** Univariate and multivariate analysis calculated with a Cox regression model between patient characteristics and the cumulative incidence of ACE.

Variables	Univariate analysis			Multivariate analysis		
	HR	95% CI	p-value	HR	95% CI	p-value
Age (Years)	1.06	0.99–1.13	0.095	1.02	0.95–1.08	0.663
aRisk factors (one or more (7) vs none (0))	24	0.02–391419	0.515			
Mean heart dose (<3 Gy (2) vs >3 Gy (5))	1.20	1.002–1.44	0.047	4.83	1.1–26	0.048
CAC score (>0 (5) or 0 (2))	34	6.6–176.8	<0.001	24	4.22–134	<0.001

Abbreviations: CAC; coronary artery calcium.

The number of ACEs were presented in the parenthesis aside for each variables.

a Risk factor; BMI (cut off value 30), Laterality, HTN, DM, Smoking, Use of anthracycline, history of heart disease.

The number of NAHDs for each disease spectrum was as follows: 23 for heart failure, 16 for stable angina, five for atrial fibrillation, and five for others, such as cardiomyopathy or valve dysfunctions. When patients were categorized into two groups with a CAC score of >0 or 0, there was no significant difference in the NAHD's cumulative incidence (10-year incidence of 5.4% and 3.8%, respectively; P = 0.44; Supplementary Fig. 2a). There was also no significant difference of NAHD risk according to the CAC score divided into 4 groups (P = 0.079; Supplementary Fig. 2b). The univariate and multivariate analyses results for NAHD are shown in Supplementary Table 1.

#### 3.2.1 Subgroup analyses

**Fig. 2:**
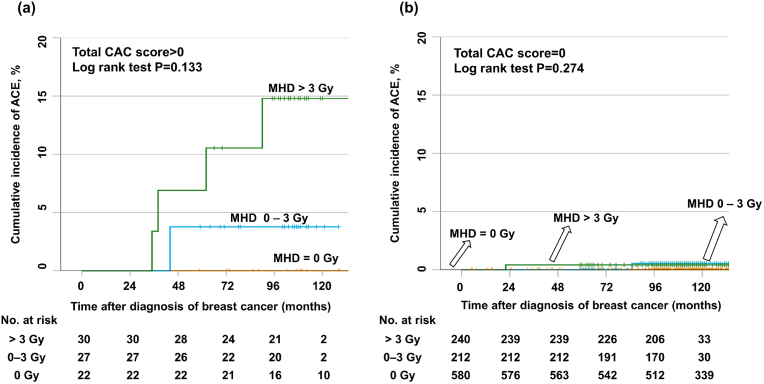
Cumulative incidence of acute coronary events (ACEs) according to the mean heart dose (MHD) in those with a total coronary artery calcium (CAC) score >0 (a) or a total CAC score = 0 (b).

The difference in the ACE risk according to the MHD was more prominent in the group with a CAC score >0 than in the group with a CAC score = 0. The ACEs' 10-year cumulative incidences in the group with a CAC score >0 were 0% [95% CI, 0–0], 3.7% [95% CI, 0.89–1.0], and 13.7% [95% CI, 0.74–0.99] in patients with an MHD of 0 Gy, 0–3 Gy, and >3 Gy, respectively (P = 0.133; Fig. 2a). In the group with a CAC score = 0, there was no significant association between ACE risk and incremental changes in the MHD (Fig. 2b).

In addition, the MHD and the 10-year cumulative incidences of ACE according to regional lymph node irradiation, tumor bed boost, and pathologic stage of breast cancer are shown as supplementary data (Supplementary Table 2).

**Fig. 3:**
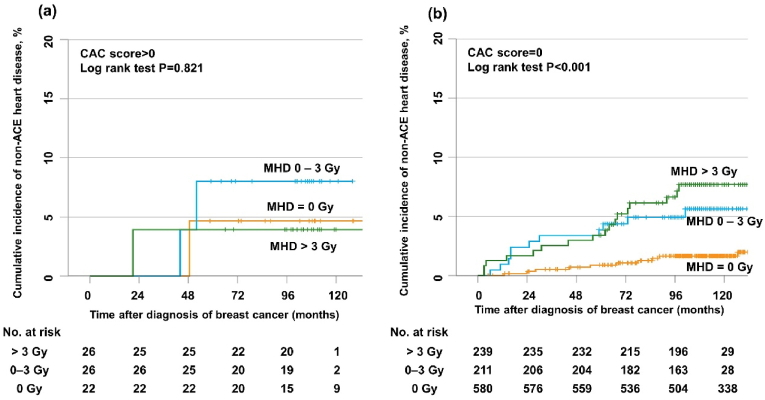
Cumulative incidence of non-acute coronary event heart disease (NAHD) according to the mean heart dose (MHD) when the total coronary artery calcium (CAC) score was >0 (a) or the total CAC score = 0 (b).

We also evaluated the risk of NAHD by MHD in the CAC score subgroups (Fig. 3a and b). In those with a CAC score = 0, the increase of NAHD's cumulative incidence seemed prominent along with the MHD (10 year cumulative incidence, 1.7%, 5.7%, and 7.1% in patients with an MHD of 0 Gy, 0–3 Gy, and >3 Gy, respectively; P < 0.001).

#### 3.2.2 Correlation between the stenosis region of ACE and the irradiated CAC region

**Fig. 4:**
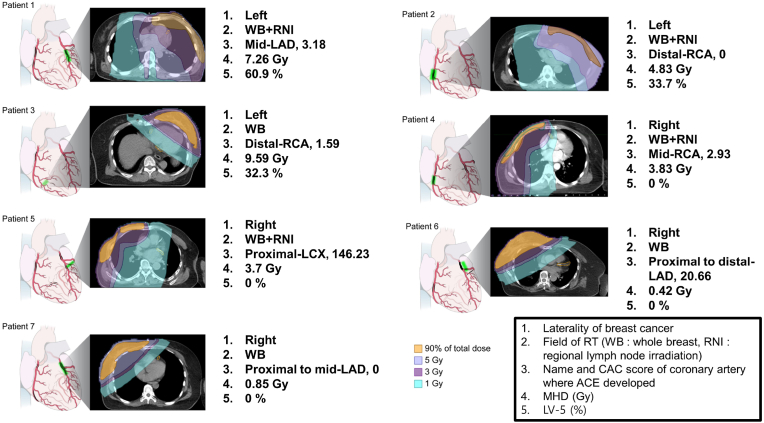
An illustration depicting the radiation fields in seven patients experiencing acute coronary events (ACEs). The specific locations where coronary stenosis was detected through coronary angiography are highlighted in green in the heart-shape illustrations (left panel in each case) and by the orange solid line in the axial view of the computed tomography (middle panel in each case). Further information about the laterality of the disease, field of radiation therapy (RT), location of the ACE, coronary artery calcium (CAC) score, mean heart dose (MHD), and left ventricle volume receiving 5 Gy radiation (LV-5) are described (right panel in each case).

Seven patients with ACEs were reviewed in depth to quantify the relationship between radiation dose and ACEs (Supplementary Table 3). The total CAC score was 0 in two patients and >0 in the others. In cases with a total CAC score >0, all ACEs occurred at the locations where coronary plaques were detected in CT scans. The median maximum and mean doses administered to the plaques where ACEs were detected were 2.23 Gy (range, 0.91–28.99 Gy) and 1.8 Gy (range, 0.7–16.65 Gy), respectively. For the two patients without calcified coronary plaques, the maximum and mean doses administered to the ACEs' locations were 2.97 Gy and 2.74 Gy in patient 2, and 1.2 Gy and 1.1 Gy in patient 7, respectively. The specific locations of the ACEs and RT fields are illustrated in Fig. 4.

## 4 Discussion

Our study re-emphasized that the CAC scores on planning CT or non-contrast chest CT correlate the risk of ACEs development in breast cancer patients. Based on our findings, we believe that patients who received even a low dose of RT might be more vulnerable to the development of ACE in the CAC score >0 group. Although the CAC score was not associated with NAHD, increased MHD was still an important risk factor for the development of NAHD even in the CAC score = 0 group. Though our data include a small number of patients with ACE, this is the first study to validate the Western studies’ findings regarding prognostic value of CAC in Asian populations [15,18].

Radiation-induced ACEs in breast cancer survivors have been investigated widely since Darby et al. first reported the dose-response relationship between radiation dose and ischemic heart disease risk in Danish and Swedish breast cancer patients [23]. Recently, the importance of evaluating individual RT doses delivered to cardiac substructures has been highlighted, and efforts are being made. For example, van den Bogaard et al. comprehensively analyzed the correlation between radiation dose to cardiac substructures and the incidence of ACEs [5]. The study used an automatic delineation tool to exclude inter-observer variability, and the cumulative incidence of ACEs per Gy of radiation to the whole heart was 16.5% (95% CI, 0.6–35.0), which was consistent with the hazard ratio of 16.3% by Darby et al. LV-5 was suggested as a significant factor for the development of ACE (HR 1.246, 95% CI 1.037–1.495, p = 0.019) in the multivariate normal tissue complication probability model, which emphasizes the importance of radiation dose to substructures. The importance of radiation dose to the left ventricle was also reported in the Korean population. Jang et al. reported that more than 60 Gy of dose to the left ventricle was associated with an increase in the cumulative incidence of ACE in patients who received concurrent chemoradiotherapy for non-small cell lung cancer (sub-distribution HR 10.06, 95% CI 1.46–69.46, p = 0.019) [24]. In the aspect of radiation to the coronary artery, one of the most recent literatures demonstrated that mean and maximum radiation doses to the left anterior descending artery were correlated with the development of major cardiac events in breast cancer patients [6]. Atkins et al. reported that LAD V15 Gy greater than or equal to 10% was associated with an increased risk of ACE in patients with non-small cell lung cancer (adjusted HR 13.9, 95% CI, 1.23–157.21, p = 0.03) [25]. The MD Anderson Cancer center group also reported that LAD V30 Gy ≥10% was associated with a higher incidence of major coronary events (p = 0.044) [26]. To reduce the radiation dose and irradiated volume of the heart, modern RT techniques, including DIBH, prone, partial breast RT, and IMRT, are being widely adopted [27], and prospective proton RT trials (NCT02603341 and NCT04291378) are expected to reduce ACEs' potential risk. However, because individual patient anatomy varies and some require an extended RT field, keeping a cardiac dose below 1–3 Gy in all patients may be difficult [28]. Furthermore, there is a paucity of data demonstrating what dose would be sufficiently low to ensure that there is no positive linear dose-response component. Generally, for breast cancer patients, the RT dose delivered to cardiac substructures tends to be low with modern RT technique, and it emphasizes the strength of our study that includes no-RT patients as a control group who absolutely received 0 Gy of radiation.

Recently, the CAC score's importance has been increasingly investigated as an ACE risk factor in patients with thoracic malignancies. Although the CAC scoring's prognostic value in predicting ACEs is unequivocal, its practical use in clinical settings has been limited, as thoracic CT scans for CAC screening are not routinely recommended for all patient populations. Therefore, investigators have used thoracic simulation-CT for RT planning to examine CAC scores and showed that patients with breast cancer were appropriate candidates [15,18]. Our multivariate analysis revealed that the CAC score and MHD were significant predictive factors for ACEs, and the CAC score's HR was the highest, consistent with the results of the two aforementioned studies. Another reason that limits an active utilization of the CAC scoring in screening ACE high-risk patients is that the manual scoring was laborious and time consuming. Recently, deep-learning-based automated CAC scoring softwares have been supported by various vendors, and Gal et al. presented a successful application of the automated CAC scoring system with 15,000 patients [18].

Though ionizing radiation to the heart is known to induce cardiovascular injury through sustained inflammation, leading to atherothrombosis after decades of RT [29], it remained unknown whether there was an interaction between preexisting coronary plaques and radiation exposure in ACEs development. In addition to one animal study that radiation accelerates atherosclerosis [30], Bogaard et al. reported clinical data that the mean radiation dose delivered to atherosclerotic plaques was the ACEs' strongest predictor in breast cancer patients [16]. Our results support these findings, as all ACEs were detected in patients who received RT, and the ACEs' specific locations in patients with a CAC score >0 were areas where calcified coronary plaques were present before RT. Although there was no statistical significance, ACEs' cumulative incidence increased according to the MHD and was prominent in patients with a CAC score >0 (Fig. 2). One Dutch study reported similar results, showing that the absolute increase in hospitalization or mortality rates due to ischemic heart disease was greater in patients with CAC deposits [17]. However, the limitation was that the patients who did not receive RT were not included in the study's analysis, and without this control group, it is challenging to properly assess the ACEs' risk resulting from RT. Therefore, the present study's novelty is that it included a patient cohort with breast cancer who did not receive RT as a control group.

The first limitation of our study is that the low incidence and small number of ACEs make it difficult to perform further subgroup analyses to clarify the risk factors or relationships between RT and coronary plaques. Considering that recent studies are emphasizing LV-5 Gy or radiation to coronary calcification as a prominent risk factor for the development of ACEs, further studies are expected. The small number of coronary events also induced the overfitting problem of the regression model used in our study. Although we converted the baseline risk factors into a dichotomous variable to overcome the problem, its statistical significance should be interpreted with caution. Another limitation of the study was that soft plaques with a radiodensity <130 Hounsfield units were not included in the analysis. Considering that soft lipid-rich plaques may exhibit a higher risk of rupture than calcified plaques [31,32], studies assessing the correlation between preexisting soft plaques and radiation exposure are expected.

## 5 Conclusions

CAC score has been successfully validated in our cohort of patients as a strong predictive factor for ACEs, but not for other types of heart disease in breast cancer patients, especially among those who have undergone adjuvant RT. Considering that the doses to the coronary artery or plaques where the ACEs developed were mostly <5 Gy and the NAHD risk increased along with the increase in MHD regardless of CAC score, best efforts should be made to keep the dose to cardiac structures as low as possible. Although further studies are required, CAC score screening on RT simulation CT scans in patients with breast cancer may provide valuable information for physicians and patients to guide individualized treatment.

## Data sharing statement

The authors confirm that the data supporting the findings of this study are available with in the article and/or its supplementary materials.

## Declaration of competing interest

There are no conflict of interests including any financial, personal or other relationships with other people or organizations within that could inappropriately influence as separate items for all authors.

## Ethics approval and informed consent

All patients included in this study provided written informed consent and the study protocol was approved by the institutional review board of Severance Hospital, Yonsei University (IRB approval number 4-2021-1342). This study was performed in compliance with the Declaration of Helsinki.

## Funding

This study was supported by a faculty research grant of 10.13039/501100008005Yonsei University College of Medicine (6-2021-0233) and 10.13039/501100003725National Research Foundation of Korea (NRF) grant funded by the Korea government (10.13039/501100014188MSIT) (No. 2019R1C1C1009359).
