# Supplementary material for: Automated coronary artery calcium scoring in patients with breast cancer to assess the risk of heart disease following adjuvant radiation therapy
Source: Breast. 2022 Jul 11;65:77–83. doi: 10.1016/j.breast.2022.07.003 (PMC9307671; doi:10.1016/j.breast.2022.07.003)
Supplement: Multimedia component 1 [file mmc1.docx]

**Supplementary table 1. Univariate and multivariate analysis calculated with a Cox regression model between patient characteristics and the cumulative incidence of non-acute coronary events heart disease (NAHD)**

| **Variables** | **Univariate analysis** | | | **Multivariate analysis** | | |
| --- | --- | --- | --- | --- | --- | --- |
|  | **HR** | **95% CI** | **p-value** | **HR** | **95% CI** | **p-value** |
| Age (Years) | 1.05 | 1.02-1.08 | 0.001 | 1.05 | 1.02-1.08 | 0.001 |
| BMI (Kg/m^2^) | 1.06 | 0.97-1.16 | 0.203 |  |  |  |
| Laterality (Left or Right) | 0.66 | 0.35-1.23 | 0.186 |  |  |  |
| HTN (Yes or No) | 2.20 | 1.19-4.07 | 0.012 |  |  |  |
| DM (Yes or No) | 2.61 | 1.10-6.18 | 0.029 |  |  |  |
| Exercise (Yes or No) | 0.12 | 0.02-0.90 | 0.039 | 0.10 | 0.01-0.75 | 0.025 |
| Smoking (Yes or No) | 1.003 | 0.14-7.29 | 0.997 |  |  |  |
| Type of surgery  (PM or MRM) | 3.92 | 2.06-7.43 | <0.001 | 2.63 | 1.24-5.56 | 0.012 |
| Anthracycline (Yes or No) | 0.62 | 0.34-1.12 | 0.114 |  |  |  |
| Anti-HER 2 treatment  (Yes or No) | 1.62 | 0.72-3.63 | 0.246 |  |  |  |
| Mean heart dose (Gy) | 1.20 | 1.11-1.29 | <0.001 | 1.13 | 1.03-1.24 | 0.009 |
| CAC score (>0 or 0) | 1.50 | 0.53-4.19 | 0.443 |  |  |  |

*Abbreviations: ACE; acute coronary event, BMI; body mass index, HTN; hypertension, DM; diabetes mellitus, PM; partial mastectomy, MRM; modified radical mastectomy, HER2; human epidermal growth receptor 2, CAC; coronary artery calcium

**Supplementary table 2. The mean heart dose and 10 year cumulative incidence of acute coronary events (ACE) according to regional lymph node irradiation (RNI), tumor bed boost, pathologic tumor size and nodal involvement of breast cancer in patients who received radiation therapy (n=511)**

| **Variables (n)** | **Median MHD (Gy, range)** | **10 year cumulative incidence of ACE, % (95% confidence interval)** |
| --- | --- | --- |
| **Right RNI** |  |  |
| Yes (n=106) | 2.11 (range, 0 – 7.43) | 1.9 (0-4.5) |
| No (n=128) | 1.13 (range, 0 – 8.52) | 1.7 (0-4.0) |
| **Left RNI** |  |  |
| Yes (n=121) | 6.97 (range, 1.28 - 14.16) | 1.7 (0-4.1) |
| No (n=156) | 5.19 (range, 0 – 12.19) | 0.6 (0-1.9) |
| **Tumor bed boost** |  |  |
| Yes (n=408) | 2.53 (range, 0.0-14.16) | 0.8 (0-1.6) |
| No (n=103) | 5.58 (range, 0.58-12.32) | 4.0 (0.1-7.7) |
| **Pathologic size** |  |  |
| 0-2cm (n=369) | 3.12 (range, 0.13 – 14.16) | 0.8 (0-1.7) |
| > 2cm (n=142) | 4.34 (range, 0 – 13.1) | 3.0 (0.1-5.8) |
| **Nodal stage** |  |  |
| N0 (n=351) | 2.71 (range, 0 – 14.16) | 0.9 (0-2.3) |
| N+ (n=160) | 4.6 (range, 0.54 – 12.32) | 2.0 (0-4.2) |

*Abbreviations: MHD; mean heart dose, ACE; acute coronary event, RNI; regional lymph node irradiation

**Supplementary Table 3. In depth review of 7 patients with ACE**

| **Variables** | **1** | **2** | **3** | **4** | **5** | **6** | **7** |
| --- | --- | --- | --- | --- | --- | --- | --- |
| **Laterality** | Left | Left | Left | Right | Right | Right | Right |
| **Age (at the diagnosis of breast cancer, Years)** | 42 | 61 | 63 | 52 | 68 | 64 | 54 |
| **HTN** | No | No | Yes | No | Yes | Yes | Yes |
| **DM** | No | Yes | No | Yes | Yes | No | No |
| **BMI (Kg/m2)** |  |  |  |  |  |  |  |
| **Smoking** | No | Yes | No | No | No | No | No |
| **Exercise** | No | No | Yes | No | No | No | No |
| **RT modality** | 3D CRT | 3D CRT | 3D CRT | 3D CRT | 3D CRT | 3D CRT | 3D CRT |
| **Prescribed RT dose (Gy)** | 50.4 | 50.4 | 59.4 | 50.4 | 50.4 | 59.4 | 59.4 |
| **RT field** | WB+RNI | WB+RNI | WB | WB+RNI | WB+RNI | WB | WB |
| **Use of cardiac medication before diagnosis of ACE** |  |  |  |  |  |  |  |
| ACEi | No | No | No | Yes | Yes | No | No |
| ARB | No | No | No | No | Yes | No | No |
| BB | Yes | No | No | No | Yes | No | No |
| Statins | No | No | No | No | Yes | No | No |
| **Time to ACE after diagnosis of breast cancer (months)** | 90.0 | 22.0 | 35.0 | 62.0 | 38.0 | 44.0 | 85.0 |
| **CAC score (Agatston score)** |  |  |  |  |  |  |  |
| LM | 0 | 0 | 0 | 0 | 0 | 50.86 | 0 |
| LAD | 3.18 | 0 | 0 | 16.11 | 143.05 | 20.66 | 0 |
| LCX | 0 | 0 | 6.36 | 0 | 4.77 | 0 | 0 |
| RCA | 0 | 0 | 1.59 | 2.93 | 146.23 | 0 | 0 |
| Total score | 3.18 | 0 | 7.95 | 19.03 | 294.05 | 71.53 | 0 |
| **Location of ACE** | mLAD | dRCA | dRCA | mRCA | pLCx | p-dLAD | p-mLAD |
| **Radiation dose (Gy)** |  |  |  |  |  |  |  |
| Mean RCA | 6.46 | 2.52 | 1.65 | 3.9 | 2.52 | 1.7 | 2.74 |
| Max RCA | 13.49 | 2.97 | 2.2 | 9.87 | 7.97 | 2.48 | 4.43 |
| Mean LCX | 5.81 | 3.65 | 1.85 | 0.6 | 1.42 | 0.6 | 0.76 |
| Max LCX | 7.32 | 4.37 | 2.15 | 0.82 | 1.91 | 0.76 | 0.89 |
| Mean LAD | 16.06 | 6.46 | 24.02 | 0.43 | 1.26 | 0.55 | 0.94 |
| Max LAD | 45.81 | 15.48 | 51.66 | 0.64 | 2.63 | 0.92 | 1.2 |
| Mean plaque dose in ACE developed coronary artery | 16.65 | N/A | 1.14 | 3.61 | 1.8 | 0.7 | N/A |
| Max plaque dose in ACE developed coronary artery | 28.99 | N/A | 1.24 | 5.66 | 2.23 | 0.91 | N/A |
| LV-5 Gy (%) | 60.9 | 33.7 | 32.3 | 0 | 0 | 0 | 0 |
| Mean heart dose (Gy) | 7.26 | 4.83 | 9.59 | 3.83 | 3.7 | 0.42 | 0.85 |

*Abbreviations: ACE; acute coronary event, HTN; hypertension, DM; diabetes mellitus, BMI; body mass index, RT; radiation therapy, 3D-CRT; 3 dimmensional conformal radiation therapy, WB; whole breast, RNI; regional lymph node irradiation, ACEi; angiotensin converting enzyme inhibitor, ARB; angiotensin receptor blocker, BB; beta blocker, LM; left main coronary artery, LAD; left anterior descending coronary artery, LCX; left circumflex artery, RCA; right coronary artery, LV-5 Gy; left ventricle volume receiving 5 Gy

**Supplementary figure 1**


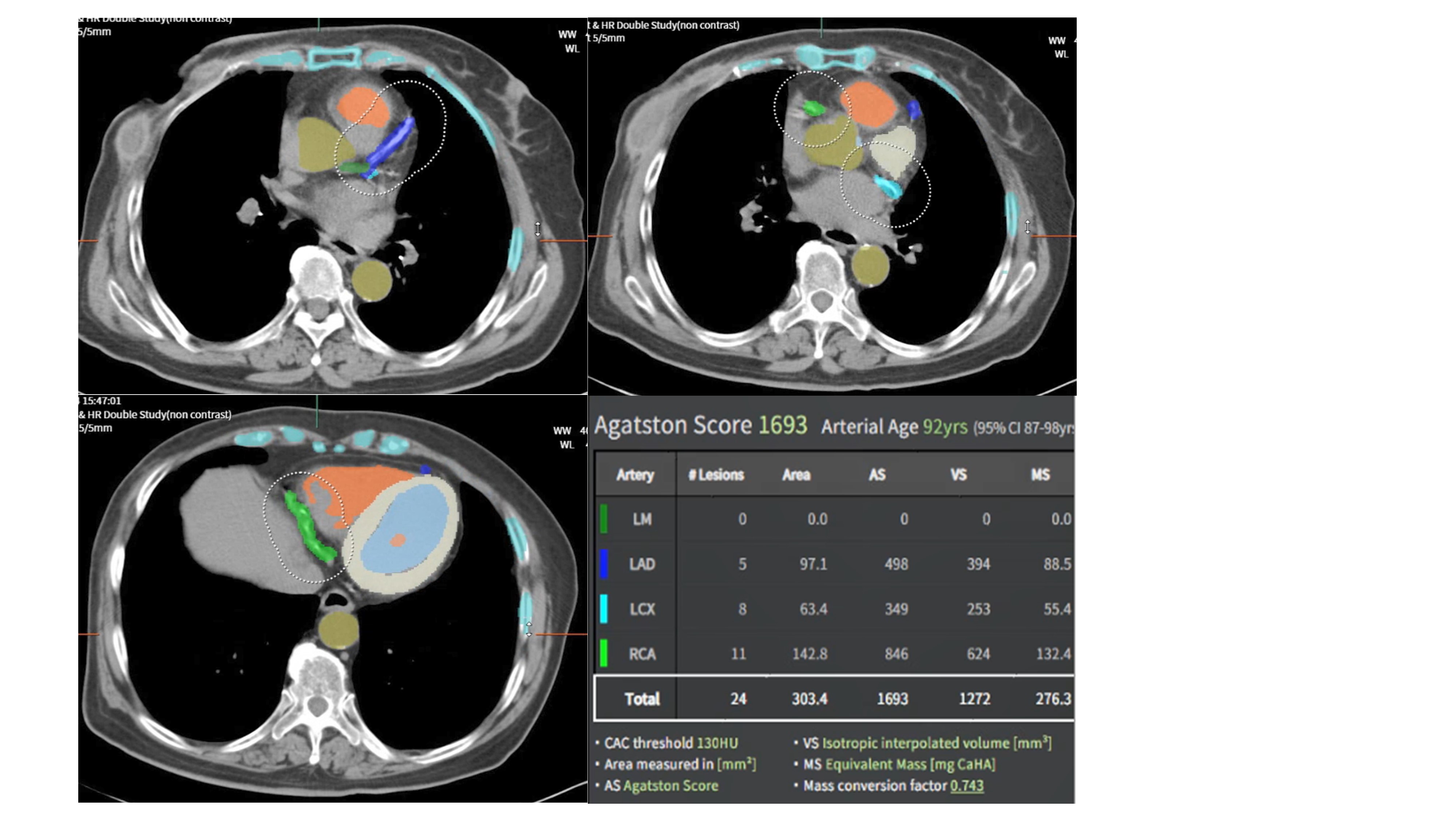


The AVIEW-CAC software can detect each cardiac substructure and calcium deposits with a radiodensity exceeding 130 Hounsfield units, presenting the results using various coronary artery calcium (CAC) scoring values, including the Agatston score (AS), a volume score (VS), and a mass score (MS). The AS for each coronary artery was calculated as the product of the lesion area (cm^2^) and the density weighting factor, and the sum of the CAC scores for each coronary artery was described as a total AS. Detected calcium in the coronary arteries is marked with dotted circles in the axial views of the computed tomography scans.

**Supplementary figure 2**


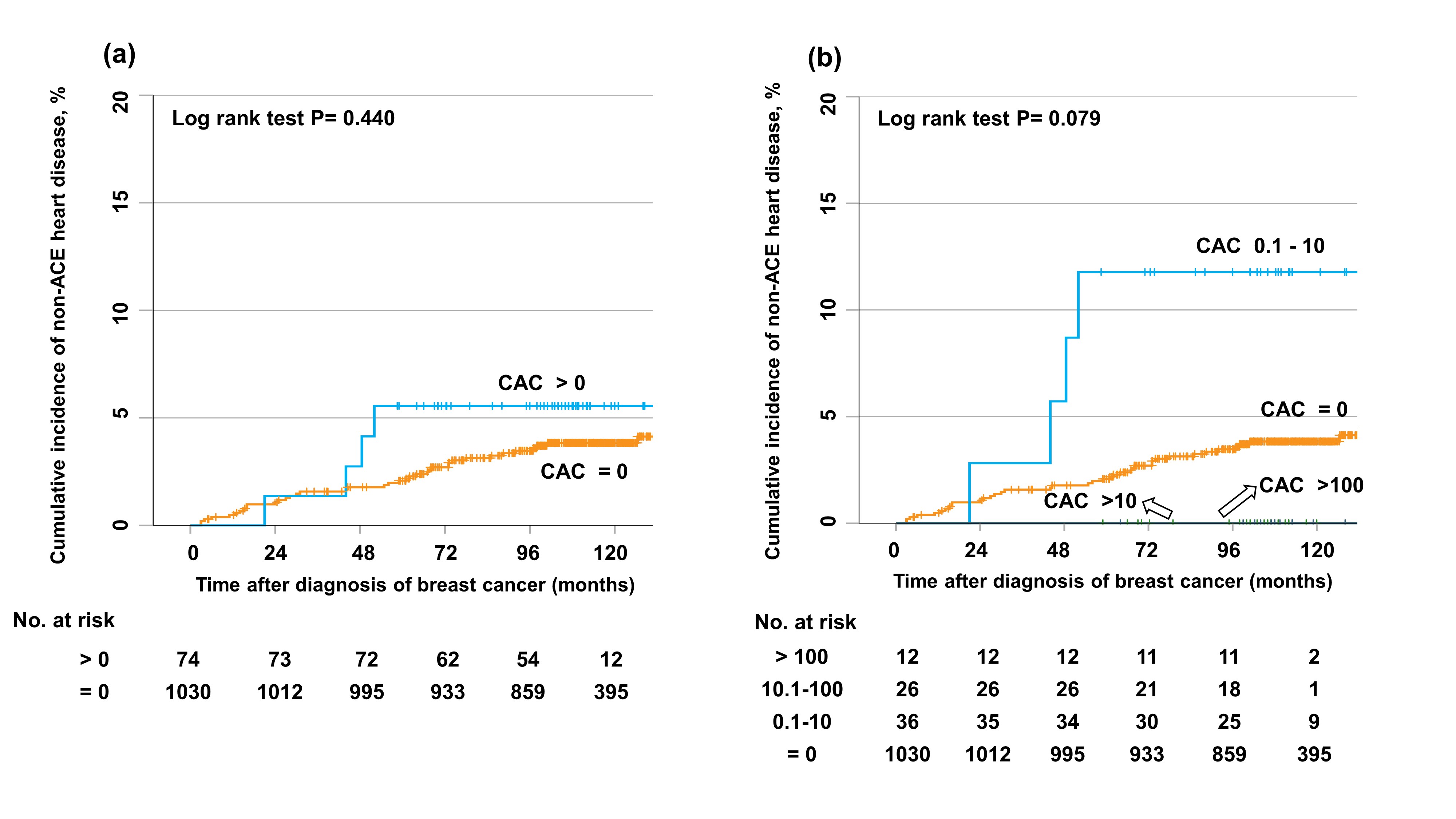


Cumulative incidence of non-acute coronary events heart disease (NAHD) when patients were categorized divided into two groups with a CAC score = 0 and > 0 (a) or when categorized into four groups with a coronary artery calcium (CAC) score = 0, 0.1–10, 10.1–100, and > 100.
